# How does single party dominance influence civil society organisations’ engagement strategies? Exploratory analysis of participative mainstreaming in a ‘regional’ European polity

**DOI:** 10.1177/0952076715581876

**Published:** 2015-05-04

**Authors:** Paul Chaney

**Affiliations:** Wales Institute of Social Economic Research and Data (WISERD), Cardiff University, Cardiff, Wales

**Keywords:** Civil society, equalities, government, mainstreaming, participation, politics, turnover

## Abstract

A raft of United Nations Treaties, European Union Directives and domestic laws oblige governments in 180 + countries to apply the Participative Democratic Model of mainstreaming equalities to public administration by involving those targeted by equality initiatives at all stages in their design and delivery. Notwithstanding Participative Democratic Model’s deeply political nature, extant work has overlooked how governing party turnover influences civil society organisations’ (CSOs) strategies. Here, this lacuna is addressed using a negative ‘extreme case study’ research design involving qualitative accounts from civil society organisations in Wales, a ‘regional’ European polity characterised by one-party dominance. The findings reveal how the absence of turnover distorts the Participative Democratic Model in relation to diverse factors including: strategic bridging, extraparliamentary politics, cognitive locks and party institutionalisation. Inter alia, the wider contribution of this analysis lies in showing the importance of turnover to effective engagement, the ‘pathologies’ associated with one-party dominance and the need for adaptive civil society strategies tailored to prevailing electoral politics and governing party turnover in liberal democracies.

## Introduction

This study is concerned with exploring the role of governing party turnover in shaping participative mainstreaming and civil society organisations’ (CSOs) strategies for engaging in the work of government. Mainstreaming is an internationally adopted approach to promoting equality. It is underpinned by a series of United Nations (UN) conventions and resolutions (e.g. CEDAW, SCR1325, SCR1888 and SCR1889), as well as European Union (EU) Directives (e.g. 2004/113/EC and 2006/54).^1^ It has also been enshrined in domestic legal codes (e.g. Equality Act, 2010).^2^ It came to prominence at the Fourth UN Conference on Women (circa 1995). Since then representatives from 189 countries have adopted the Participative Democratic Model (PDM) of mainstreaming as set out in the Beijing Declaration and Platform for Action. Originally conceived as an approach to gender equality (cf. Beveridge and Shaw, 2002; Woodward, 2008), its application has broadened to incorporate a full range of characteristics (e.g. age, disability, faith, ethnicity and sexual orientation). It is concerned with embedding equality into all stages of public policy making. Crucially, the participative model is based on engaging groups targeted by equality objectives in their design, development and implementation.

Notwithstanding its prominence, a burgeoning literature attests to the difficulties of translating mainstreaming principles into practice (cf. Bacchi and Eveline, 2010; Beveridge and Nott, 2002; Caglar, 2013; True and Parisi, 2013; Woehl, 2011). As Mosesdottir and Erlingsdottir (2005: 525) observe, ‘very few countries have managed to develop a well-functioning institutional framework around the mainstreaming method’. The UN concurs. Its appraisal of progress since the Beijing Declaration concluded, ‘many gaps and challenges remain in guaranteeing … full and equal participation in decision-making in all stages’ (UN, 2010: 37). Mainstreaming is intimately concerned with power and agenda setting – and the challenging of exclusive norms and the exercise of power by elites unrepresentative of social diversity. Yet, curiously, existing scholarly work has tended to overlook its deeply political nature. Instead, past studies have tended to regard government as a largely non-party-political policy actor. Moreover, extant research has paid scant attention to the role of electoral politics and governing party turnover (‘turnover’ here refers to the rate at which the party holding government office is voted out in state-wide elections and replaced by a rival). It is a significant knowledge gap, one that the following discussion addresses.

This study follows a negative ‘extreme case study’ design (for a full discussion, see Methodology section) and focuses on Wales; a rare and striking example of a liberal democratic system where governing party turnover is absent – for the country has experienced single-party dominance throughout the post-war period. The findings are based on qualitative data analysis of interviews with civil society policy actors. The specific focus on CSOs is appropriate here because state–civil society relations lie at the heart of participative mainstreaming. Thus, the Beijing Declaration and Platform for Action (UN, 1995) asserts: ‘civil society cooperation with Governments [is] important to the effective implementation and follow-up of the Platform for Action’ (UN, 1995, Article 20). In conceptual terms, mainstreaming is based on criticality and engagement between state and *non-*state actors. This precludes both the inclusion of civic society organisations (because of their connection to the state) – and the private sector (which is eschewed owing to its concern with capital accumulation/the profit motive, rather than representing the policy claims of those with ‘protected characteristics’).

As the following discussion reveals, this study makes three original contributions: (i) it synthesises diverse strands of social theory and applies them to the PDM as part of an exploration of how single-party dominance influences CSOs’ strategies; (ii) it presents the core argument that, although often overlooked, governing party turnover matters to the PDM for it shapes the political opportunity structures and attendant CSO strategies for engagement in the work of government; and (iii) it emphasises that CSOs’ need to employ an adaptive model of policy engagement suited to the prevailing electoral politics and governing party turnover in liberal democracies. The findings are presented in relation to four themes emerging from the data analysis. To guide the reader, these are now briefly summarised and returned to in detail in the Findings section.

1. In the first part of the findings, social theory in relation to the Collective Interest Model and strategic bridging is applied to the interview data. This reveals how OPD skews the political dynamic for participation in the work of government. Not least it shapes group incentives – as well as whom to engage amongst political elites.
2. Subsequently, drawing on theory on action repertoires (or, the means by which CSOs advance their policy demands), attention centres on the way turnover distorts the political dynamic for participation. Interviewees allude to the mixed impact of OPD. Some view the absence of turnover as a pathology, limiting their policy input. Others see it as a spur to action, a catalyst for new repertoires to be employed in order to place policy demands on those in power.
3. Next, theory on framing and ‘cognitive locks’ again underlines how turnover shapes engagement. Here the notion of ‘intellectual path dependency shows how, under OPD, CSOs are found to be more constrained in their use of language. Rather than a free exchange of ideas, interviewees speak of deliberately advancing claims in ways that align with dominant party thinking.
4. Finally, theory on policy networks, CSO alliance-building and neo-corporatism reveals how the disproportionate power of the dominant party distorts collective action and leads to ‘defensive’ networking amongst CSOs in order to counter the hegemony of the governing party.

Attention first turns to a summary of the literature on mainstreaming and one-party dominant systems. In turn, this is followed by an outline of the research context and methodology, followed by the findings.

## Equalities mainstreaming

Mainstreaming has been described as ‘one of the most rapidly adopted, progressive social justice-oriented initiatives endorsed by the international community in the modern era’ (Chaney and Rees, 2004: 24). The Council of Europe (2003: 7) defines it as: ‘the (re)organisation, improvement, development and evaluation of policy processes, so that an equality perspective is incorporated in all policies at all levels and at all stages, by the actors normally involved in policy-making’. Two broad approaches can be identified (Barnett Donaghy, 2003; Nott, 2000), the ‘participative-democratic’ (PDM) and ‘expert-bureaucratic’ models. The latter is a technocratic method reliant on experts. In contrast, the former (and subject of this paper) is predicated on exogenous groups advancing equality claims on government through active participation and engagement with civil society (Lister and Carbone, 2006). As Debusscher and Van der Vleuten (2012: 326) observe, ‘mainstreaming is constructed, articulated and transformed through discourse, policy-makers carry the responsibility to push […] equality further by involving civil society and individual activists promoting […] equality’. ‘Participation’ and ‘engagement’ here can be defined as the full range of formal and informal means employed by individuals and groups to influence the aims, scope, design and implementation of public policy (Hogwood and Gunn, 1984). These include protest, boycott, lobbying, petitions and consultation responses.

As noted, engagement with equalities groups in civil society is a core requirement placed on government in a raft of international treaties and directives. It is a fundamental tenet of the Beijing Declaration: ‘full participation on the basis of equality in all spheres of society, including participation in the decision-making process and access to power, are fundamental for the achievement of equality, development and peace’ (UN, 1995: 3). Subsequently, it has been codified in no less than 10 UN resolutions^3^ (the most recent being in 2010 – Resolution A/RES/65/191) – as well as a series of EU directives including Council Directives on equal treatment between persons irrespective of: racial or ethnic origin (2000/43/EC); religion and belief, disability, sexual orientation and, age (Directive 2000/78/EC); as well as Sex (Art. 157 TFEU [ex. Art. 141 EC]; Dir. 2006/54/EC and Dir. 2004/113/EC). It is also detailed in governments’ human rights obligations (e.g. Charter of Fundamental Human Rights, Art. 21(1) and (2), Art. 23; European Convention on Human Rights (ECHR) and Fundamental Freedoms, Art. 14; Protocol 12). Furthermore, it is set out in domestic equalities law. Thus, for example, Fyfe, Johnston Miller and McTavish (2009: 211) conclude, ‘the Equality Act (2006) has firmly positioned gender equality mainstreaming within UK public policy’. This is evidenced by the requirement on listed authorities to report progress on mainstreaming the public sector equality duty under the Equality Act (2010; see Equality and Human Rights Commission, 2012).^4^

The foregoing political and legal imperatives have ensured that mainstreaming has entered the lexicon of policy reform (True and Mintrom, 2001). They have also underpinned wide-ranging institutional measures in government and beyond (such as the creation of equality policy units and application of policy tools such as impact assessments – see Jasquot, 2010; UN, 2010). According to a one UN survey, 165 member states had some form of ‘national machinery’ for mainstreaming by government (Inter-Agency Network on Women and Gender Equality, 2005: 61). However, whilst a limited number of studies have underlined the role of political context in shaping mainstreaming practice – including the effects of: a hostile political climate (Nott, 2005), political interpretation and enforcement (Payne, 2011), and the presence of ‘woman-friendly’ administrations (Kim and Kim, 2011), the majority of extant analysis has tended to ignore electoral politics, instead regarding government as a curiously non-party-political entity.

## Governing party turnover and one-party dominant systems

Governing parties’ disposition towards civil society varies across the political spectrum; with some favouring authoritarian, ‘top-down’ approaches to public administration and others coproduction, participation and dialogue (cf. Heritier and Rhodes, 2011). Accordingly, the type of party elected to form the executive clearly matters to the PDM and understanding the patterns and processes influencing CSOs’ engagement. Such factors also provide an insight into the health of a given democracy. Thus, as Mair (1996: 84) observes, one can ‘trace sources of problems of legitimacy and stability of regimes back to the character of their party systems’. This view is consonant with Huntington’s seminal study (1991) that emphasised turnover as a key predictor of democratic maturity. Inter alia, it shapes the circulation of the political elite (Mosca, 1938), openings for new political leaders, the prevailing political opportunity structures for social interests and the scope for introducing innovatory practice in public policy and administration.

Moreover, as Jackson (1994: 270) observes, the *absence* of turnover undermines democracy. A key gap in the academic literature is whether such assumptions are supported by empirical data on mainstreaming and CSOs’ policy engagement. Existing work suggests that a fragmented party system (with comparatively high rates of governing party turnover) will offer greater political opportunities for CSOs to advance policy claims on those in power. This is because the attendant political and electoral vulnerability of parties creates a need to form coalitions resulting in party elites’ greater flexibility in the face of popular demands (Saward, 2006). In contrast, strong parties in less fragmented systems are better placed to disregard civil society claims. To summarise, the distortion of the civil-society nexus implied by the literature on one-party dominance (OPD) is founded on (1) diminished legitimacy and accountability (dominant parties have less electoral vulnerability and can ignore/resist popular demands and impose their agendas at will); (2) the foregoing limits the political opportunity structures for social interests; (3) there is less circulation of political elites heightening the problem of veto players and (4) influence of the dominant party extends into civil society where criticality may be compromised as CSOs are reluctant to forego state funding and patronage.

As Greene (2010: 155) explains, one-party systems are ‘“odd ducks” that incorporate genuine electoral competition with the absence of turnover’. International examples include Japanese politics 1955–2009 (Inoguchi, 2005) and South African politics 1994–present (cf. Bénit-Gbaffou, 2012). Far from being an obscure and unlikely eventuality, all liberal democratic systems have inherent potential for episodes of OPD. The value of studying an OPD system – as in the present case, is that it effectively constitutes a control experiment and reveals the role and importance of turnover to participative democratic mainstreaming. There are varying definitions of what constitutes OPD. In his classic text, Duverger (1954: 308–389) refers to a dominant party as one whose ‘influence exceeds all others for a generation or more [ … whose] doctrines, ideas, methods – its style, so to speak, coincide with those of the epoch’. In like fashion, Butler (2009: 159) notes that OPD:

has been used to refer to the protracted electoral and ideological dominance of a one party in a representative democracy. It requires, but suggests more than, a series of electoral successes. OPD implies institutions that translate electoral success into political power; the capacity to attract support from substantial electorates over an extended period; the presence of a unifying historical project; and the ability to dominate the policy agenda of a country.

Furthermore, Greene (2010: 823) offers a number of defining characteristics of OPD systems. These include:

holding the premiership, [and] at least a plurality of legislative seats; a longevity threshold – typically a four election/20 year span; electoral competition must be meaningful (this entails … a legislature that cannot be dismissed by the executive and who are chosen through regular popular elections, opposition forces that are allowed to form independent parties and compete in elections, and the incumbent does not engage in outcome-changing electoral fraud).

As the following discussion reveals, when OPD systems emerge, they distort the civil society–state nexus, potentially subverting the beneficial democratising elements of criticality and resource exchange seen in other liberal democracies. In such instances, as Giliomee and Simkins (1999: 340) note, ‘the vital elements of democracy, namely genuine competition and uncertainty in electoral outcomes, are removed in a process that is self-sustaining’. The way that this impacts on civil society engagement with government is a key knowledge gap. It matters because, it is a core tenet of equalities mainstreaming – as well as pluralist theory (Dahl, 1961). Both underline how exogenous civil society interests perform a pivotal role through knowledge transfer, service delivery, and critical engagement as part of the wider processes of agenda setting and holding government to account. As a result, OPD has the potential to introduce a range of obstacles to participative mainstreaming – and, crucially, illuminate the role of governing party turnover. Prior to discussing these, attention first turns to the research context.

## Research context

As Rodriguez-Pose and Gill (2003: 334) note, ‘a devolutionary trend has swept the world [ … involving widespread] transference of power, authority, and resources to subnational levels of government’. A burgeoning literature attests to the fact that this is not a uniform process (Krossa, 2011). The rise of meso- or ‘regional’ government across Europe and beyond is characterised by contrasting powers and levels of autonomy being transferred to regions within UN-recognised unitary states (Heritier and Rhodes, 2011). The UK is no exception. State decentralisation (or devolution) in 1998–1999 moved the UK further down the constitutional path towards being a (quasi-)federal state. The (re-)establishment of legislatures for Scotland, Wales and Northern Ireland (circa 1998/1999) has accentuated divergence of the prevailing legal and policy frameworks applying to CSOs in the four constituent UK polities. It has also resulted in contrasting institutional arrangements for engagement with government (cf. Birrell, 2012). Crucially, it has meant civil society–state relations have been shaped by territorially specific patterns and processes of meso-level politics.

As in other international contexts, the UK has adopted an asymmetrical model of devolution. Although initially possessing the weakest set of powers, the Welsh ‘settlement’ is the one that has seen most change. No less than three devolution statutes have been passed by Westminster since 1998, each transferring further significant policy responsibilities to Wales (including health, housing, social services, economic development and education). Moreover, the National Assembly for Wales gained primary legislative powers in 2011 and the latest Act (in 2014) set out tax-raising powers. This is an ongoing process. The UK government has committed to further change following Commission on Welsh devolution in 2014 that recommended the devolution of further competencies, including policing, youth justice and devolution in the courts system and judiciary. These developments place regional government in Wales broadly on a par with other legislative regions across Europe and beyond.

The present locus of enquiry is propitious because, over the past 150 years, Welsh politics has been characterised by OPD. Throughout the second half of the 19th century, Wales experienced one-party domination under the Liberal Party (Morgan, 1981). Following a brief interregnum, since 1945 the Labour Party has been the preeminent political force (Hopkin, Tanner and Williams 2001; McAllister, 1980). Having secured 58.6% of the vote in the 1945 election, it has gained a majority of Welsh Members of Parliament in all subsequent ballots (and often an absolute majority of Welsh votes). Notably, in electoral terms, the other main UK state-wide party, the right-of-centre Conservative Party, has fared worse in Wales than in England at every general election since the 1800s. Moreover, following devolution, Labour’s pre-eminence is underlined by the fact that it has always gained the largest share of the vote. In 1999, it won 28 of the 60 seats in the National Assembly for Wales (polling 37.6% of the vote, 9.4% more than their nearest rivals). In the next three ballots, the party won exactly half of the seats in the legislature – the same as its three main rivals *combined* (with 40% of the vote in 2003, 32.2% in 2007 and 36.9% in 2011). This record has ensured that Labour has continuously held government office since the National Assembly for Wales was created in 1999.^5^

## Methodology

The following discussion is based upon qualitative semistructured interview data gathered from a series of studies spanning the period 1999–2015.^6^ The analysis is aligned with phenomenological and interpretive paradigms. The emphasis is on constructivist analysis; specifically, understanding policy actors’ social constructions of interaction with government (see, e.g. Cassell and Symon, 1994: 2). Thus, as Denzin and Lincoln (2000: 8) observe, qualitative exploratory case study analysis ‘implies an emphasis on the qualities of entities and on processes and meanings that are not experimentally examined or measured in terms of quantity, amount, intensity, or frequency’. Accordingly, the quotations presented in the findings below are illustrative of key themes emerging from the data where the aim is to explore and examine policy actors’ views and experiences.

The adopted methods provided a singular longitudinal perspective of the entire period of meso-governance since constitutional reform in the UK in 1998–1999. As noted, the UN policy framework (UN, 1995) states that the PDM of mainstreaming is predicated on government/state engagement with civil society. Accordingly, a purposive sample of 103 interviews was conducted with policy actors from CSOs (managers, coordinators and project workers), representing a cross-section of ‘protected characteristics’. The sample comprised seven CSOs concerned with gender equality, six ethnicity, eight disability, six age, two sexual orientation, three faith and five generic equalities. In turn, the number of interviewees for each characteristic broadly reflects the number of representative CSOs for each group as listed in the official third sector register of CSOs (Wales Council for Voluntary Action, 2014).

The interviews were based on an interview schedule consisting of core questions developed from leading texts on mainstreaming (see ‘References’). The use of a basic interview framework – or standardised set of research stimuli, allowed comparison of participants’ accounts. Semistructured interviews were particularly suited to the present task because they permitted the use of probes and secondary, supplementary questions (King and Horrocks, 2010). This boosted reliability of the data by enabling the exploration and clarification of the issues and experiences described by participants. The theoretical framework underpinning the schedule was participative democratic mainstreaming – as well as the literature on deliberative democracy (cf. Elster, 1998; Gutman and Thompson, 2004). Topics covered included action repertoires, mobilising structures, networking, communication, information, awareness, resource issues, access, influence, knowledge, skills and human capital. Interviews were transcribed and analysed using appropriate software. A deductive approach to analysis was used in order to allow the data to ‘speak’ for themselves without the imposition of predetermined coding categories (Boyatzis, 1998). Care was taken to minimise interviewer bias through use of a general interview schedule. Moreover, care was taken to uphold objectivity when asking supplementary or follow-up questions. This methodological framework allowed triangulation, in other words, comparison of emerging themes and issues across ‘protected characteristics’ (i.e. between CSOs representing women, disabled people, ethnic minorities and so on).

In terms of case study selection, Wales was chosen because it provides a rare and striking example where governing party turnover is absent. It is a liberal democratic system that has experienced single-party dominance throughout the entire postwar period. It is therefore ideally suited to an exploratory, negative ‘extreme case study’ research design (Gerring, 2004; Jahnukainen, 2010: 89). This is used to address the principal research question: ‘How does single-party dominance influence CSOs’ engagement strategies’?^7^ As the research methods literature attests, an ‘extreme case study’ is apposite ‘when the objective is to achieve the greatest possible amount of information on a given problem or phenomenon, [in such circumstances] a representative case or a random sample may not be the most appropriate strategy. This is because the typical or average case is often not the richest in information. Atypical or extreme cases often reveal more information’ (Flyvbjerg, 2006: 222). Seawright and Gerring (2008: 298) concur with this assessment:

the extreme case approach to case study analysis is therefore a conscious attempt to maximize variance on the dimension of interest, not to minimize it … is a purely exploratory method—a way of probing possible causes or possible effects of in an open-ended fashion.

**Figure 1.**
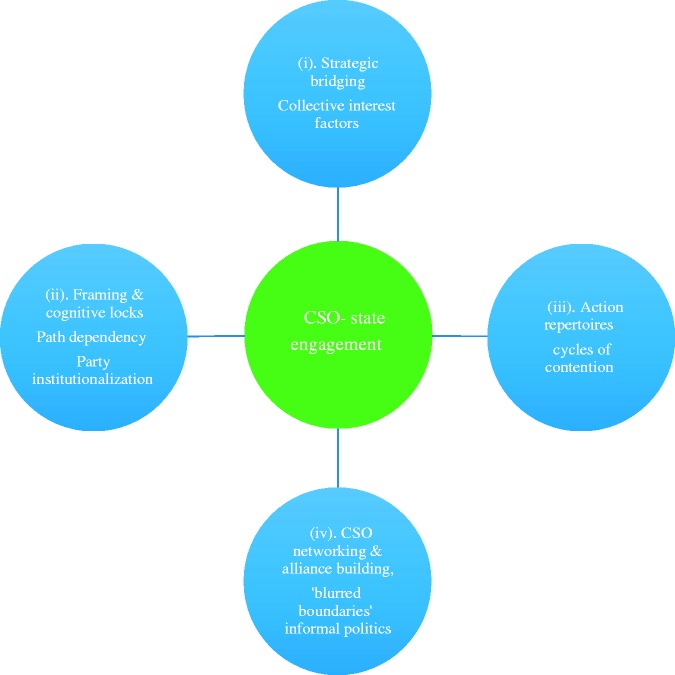
Turnover-related factors influencing civil society organisations’ engagement strategies.

Attention now turns to the research findings. Four sets of factors – or ‘spheres’ were identified by interviewees as influencing how governing party turnover affects the PDM of mainstreaming under single-party dominance^8^: collective interest representation and strategic bridging, action repertoires, framing and cognitive locks and CSO networking and alliance building (Figure 1). Each is discussed in turn.

## Research findings: The influence of governing party turnover on CSOs’ strategies

### Collective interest and strategic bridging

This first section of the findings reveals how OPD skews the political dynamic for participation in the work of government. Not least it shapes group incentives – as well as whom to engage amongst political elites. As Payne (2011: 528) cogently observes, mainstreaming has both coercive and voluntary elements. Governments may variously be seen to ‘coerce’ CSOs into policy engagement through the imposition of structural arrangements, reporting mechanisms and resource dependency. Whereas on the voluntaristic side of the equation, CSOs’ are proactive, independent policy actors concerned with placing their own demands on government.

Interviewees’ comments suggest OPD shifts the accent towards the coercive side of the equation. This can be seen with reference to the Collective Interest Model (Finkel et al., 1998: 39) which outlines three categories of group incentives for engaging with those in power: (1) ‘high levels of discontent with the current provision of public goods by the government or regime’, (2) the belief ‘that collective efforts can be successful in providing desired public goods’ and (3) the belief that CSOs ‘own participation will enhance the likelihood of the collective effort’s success’. Each of these resonates with equality claims making under the PDM and each is mediated by turnover. In turn, this skews the overall political dynamic for CSOs’ participation in the work of government. Further understanding of precisely how this happens can be gained using the concept of ‘strategic bridging’ (Tilly, 1978: 125–133). This is a concept highlighting individual agency and engagement between policy actors in civil society and parliamentarians (see also Gamson, 1975). Crucially, CSOs’ collective interests are shown to be modified by turnover. This shapes targeted lobbying of individual ministers and backbenchers in order to advance equalities claims. This is because, when participating in government policy making, CSOs are faced with the strategic choice of *whom* to engage amongst a given parliamentary cohort. This is where OPD creates a singular political dynamic for participative mainstreaming. Notably, the present research data show how it reduces the value of alliance building with opposition party members. The reason for this being that they are ‘devalued’ – or viewed as less influential owing to the fact that (compared to ‘regular’ turnover liberal democracies), they are less likely to hold future government office.

Thus, when asked about how OPD affected the targeting of parliamentarians, one respondent said

when I started [to work for the CSO] a colleague said to me ‘you’ll learn – opposition parties might be more willing to cooperate but go after Labour [i.e. the dominant party]’ … and I was all principled and that … No I’ll speak to them all – but he was right … Obviously we still try and work with the others [opposition parties] but, in terms of pay-off, the priority has to be Labour … It just has.

The associated pattern of CSO (dis-)engagement with opposition parliamentarians has worrying implications. Not least, it may further comprise attempts at PDM (– and democracy more widely) over future electoral cycles – for CSOs’ continued emphasis on engagement with the dominant party further strengthens its power and influence at the expense of opposition parties. Allied to the foregoing, in terms of strategic bridging, the present data also reveal that turnover not only shapes which parties CSOs engage – but also whom they engage in a given parliamentary cohort. Specifically, governing party turnover affects participatory mainstreaming through the existence of veto players. These are parliamentarians who, by virtue of their office, may block exogenous claims making by CSOs on issues like equalities (Tsebelis, 1995). Veto players may be unreceptive to mainstreaming claims for a variety of reasons including political ideology, *laissez faire* orientation, or discriminatory attitudes. Crucially, their influence is heighted under OPD as they are less likely to be replaced by a change of government following an election. Instead, they have enduring influence and may effectively block CSO claims over successive election cycles. Thus, for example, the chief executive of a lesbian, gay and bisexual peoples’ CSO alluded to how a prominent politician had rejected policy demands from the organisation when holding one ministerial portfolio – and had subsequently frustrated the CSO’s lobbying on a another matter when later appointed to a different ministry: ‘our hearts sank when we heard [named minister] had got it [the health portfolio in a cabinet reshuffle] … we had a clear indication of the way things were likely to go from the last time … ’

Overall, the present findings align with the wider international experience of lobbying (e.g. Parés, Bonet-Martí and Martí-Costa, 2011); yet, hitherto the connection with PDM has not been stated. It is worth reflecting on the practical implications of this finding. It suggests that CSOs need to adapt their strategic bridging so that their targeted lobbying of individual ministers and backbenchers is aligned with governing party turnover – and where appropriate, *compensates* for OPD by deliberate engagement with weaker opposition parties. Failure to do this will reduce the value of alliance building with opposition party members, increase the power of veto players in dominant parties – and further the party politicisation of claims making. In turn, this will lead to a downward spiral, thereby increasing likelihood of OPD over time and bringing with it associated negative consequences for the PDM.

### Action repertoires

This aspect of the findings provides further insight into how turnover skews the political dynamic for participation. As the following reveals, interviewees variously allude to OPD as a pathology limiting their policy input and as a catalyst for new repertoires to place policy demands on those in power. Thus, under the PDM of mainstreaming once CSOs decide to advance equality claims on those in power, they must choose the method or means by which to advance their policy demands. The options constitute CSOs’ ‘action repertoires’ (see Tilly, 1995: 42). The full gamut of claims-making methods open to CSOs is diverse. At one end of the spectrum lies protest and civil disobedience, whilst more bureaucratised means (such as policy lobbying and consultation work) are at the other.

Crucially for participative mainstreaming, CSOs’ action repertoires shift over election cycles and are shaped by the type of party holding government office. A change of governing party is often the catalyst for new repertoires to be employed – or for existing ones to be revised. It is a cyclical view of mainstreaming participation, whereby repertoires are shaped in the context of peaks or cycles of protest activity or ‘cycles of contention’ (Tarrow and Tilly, 2001). The present findings suggest that governing party turnover determines the degree to which party institutionalisation sets in. This can be defined as ‘the process by which a practice or organization becomes well-established and widely known, if not universally accepted. In consequence actors develop expectations, orientations, and behaviour based on the premise that this practice or organization will prevail into the foreseeable future’ (Mainwaring and Scully, 1995: 4). It is a situation that resonates with the notion of ‘path dependency’ in (neo-)institutionalist theory. As Pierson and Skocpol (2002: 6) explain, this refers to: ‘the dynamics of self-reinforcing or positive feedback processes in a political system … [involving] mechanisms that reinforce the recurrence of a particular pattern into the future … ’

Interviewees offered contrasting views of the impact of OPD on CSOs’ action repertoires. Some indeed supported the idea of ‘path dependency’ and alluded to how party institutionalisation under OPD had a stifling effect on participative mainstreaming. Accordingly, one policy officer

it can get a bit stale if you know what I mean … the best way to be heard is to link-in with Labour [Party] ‘spads’ [special advisors] to ministers and senior officials … [but] it can get a bit like Groundhog Day,^9^ mind … 

In contrast, others underlined how the diminished political opportunity structures under OPD had a galvanising effect on engagement based on the necessity for a broad repertoire of protest and mobilisation. For example, one said ‘you have to work round it if you like … for us we’ve tried to engage young people [and] encourage them [to use] social media campaigns … it’s just that thing of sort of trying to get round that political roadblock’. Another opined,

We’ve tried all the conventional ways, consultations and the like … it always seems to fall on deaf ears as it were, either they don’t hear us or they don’t want to hear us. Most likely both! … after the last one [i.e. election] and they [the Labour Party] got in, we said this has gotta change … we’ve got to step it up, [and] that when it started really [series of campaigns]. I have to say, I think we were slow really … We were a bit snail-like in realising they [Labour government] are going nowhere [i.e. they’ll remain in office over election cycles] it forces you to reconsider … [To] up the ante.

These findings resonate with the wider participation literature (e.g. work in Catalonia; see Font and Galais, 2011). In terms of the practical implications for CSOs, the current findings show that even when denied the catalysing effect of regular government turnover, CSOs need to be proactive, vary their action repertoires and employ the full gamut of claims-making methods. This is to ward against party institutionalisation, maximise criticality and advance claims making on government in order to challenge existing power relations in policy making.^10^

### Framing and cognitive locks

Drawing upon theory on policy framing and ‘cognitive locks’, the following furthers understanding of how single-party dominance shapes civil society engagement. Specifically, under OPD, ‘intellectual path dependency’ asserts itself and CSOs are found to be more constrained in their use of language. Rather than a free exchange of ideas, interviewees spoke of deliberately framing claims in ways that aligned with dominant party thinking. Frames here are a psycholinguistic means of ‘selecting, organizing, interpreting and making sense of a complex reality to provide guideposts for knowing, analysing, persuading, and acting’ (Rein and Schon 1993: 146). Social theory (Snow and Benford, 1992: 137) underlines how CSOs can ‘strategically frame’ issues such as equalities policy demands in order that they fit – or resonate – with the dominant frames held by other policy actors, including government. As Pollack and Hafner-Burton, (2000: 435) explain, those in power are ‘more likely to adopt new frames that are resonant, rather than in conflict, with their existing dominant’ frames’. A surface reading might suggest that OPD would make CSOs’ task easier. Ergo, in a ‘regular’ liberal democratic system government is constantly changing over election cycles, therefore one might expect a need for CSOs to consistently vary framing practices to align with the political complexion of those in power. However, respondents alluded to the specific challenges posed by OPD. In the present case study, the Labour Party has placed strong rhetorical emphasis on a policy discourse of ‘traditional’ socialism (in contrast to more centrist interpretations of social democracy held by the UK Labour Party at Westminster). For example, it has featured the tropes of mutuality, equality of outcome and redistribution (Chaney and Drakeford, 2004). Accordingly, one interviewee alluded to how they had deliberately linked equality of opportunity claims with (government) aspirations for tackling income inequalities. This was done in order to align the CSO’s framing with one of the dominant frames in the governing party discourse. She recalled how they had pressed a minister and his team on gender budgeting with limited success. Subsequently, she alluded to how:

We had this rethink … OK, we know they were [i.e. government minister] not overly keen … [That is an] understatement! I think there was a concern with [the potential cost of] equal pay claims in local government, anyway … we thought [if] we pitch it in terms of the economic aspect – ‘Clear Red Water’, Classic Labour – all that [references to a notable First Minister’s speech stating the party’s socialist governing principles] and the tackling poverty agenda [a key policy goal of the governing party] … and it was after that there was the pilot [scheme] … its easy after the event I know – but I do think the change of language on our part helped”.

An allied finding is the way that governing party turnover – and thus party institutionalisation – may shape mainstreaming practices through ‘cognitive locks’. The latter represent what Forestiere and Allen (2011: 381) term ‘intellectual path dependency in policymaking’. Once established, they become a guiding set of ideas or ‘ideological mantra to be repeated and applied no matter … [what] the actual conditions of a situation’ (Blyth, 2002: 229). They are particularly evident under one-party dominant regimes because change or reversal of government policies as a result of exogenous pressure – what Hirschman (1970: 30) calls ‘voice’ – is limited or absent. As noted, in the present case study, the dominant Labour Party has long espoused a socialist ideology and a generally rejected mixed economy approaches to welfare involving the private sector. Against this background, the manager of a disabled people’s CSO gave an example of an ideologically grounded cognitive lock:

we asked her [the minister] … if the only way to ensure the service was to contract out [i.e. to a private company] rather than face a total loss [of provision] … our members would back that … she wasn’t having it. No way … We were told in no uncertain terms there was no place for *that* route … we [the CSO] weren’t happy with it – but we’d thought about it pragmatically and felt it’d be better than nothing … they were *not* having it’.

The present findings also reveal the party politicisation of engagement. Thus, interviewees underlined how they were obliged to negotiate the challenging and singular positional politics that characterise a one-party dominant system. Compared to ‘regular’ liberal democracies, the key difference is the absence of turnover means CSOs are consistently pitted against the same party, thereby strengthening the appearance of partisanship – wherein criticism of government may become conflated with criticism of party *qua* party. It is a viewpoint captured by the following interviewee:

My personal view is the fact that it is a one party state actually does create quite a sterile environment in terms of policy-making. It very much feels that at times to challenge a policy is very much to challenge the [Labour] Party, and to challenge the hegemony of the Welsh Labour Government.

Another policy officer said: “it can get a bit ‘political’ at times … someone from the [policy network] said to me … ‘why are you giving Labour a hard time’? [i.e. the viewpoint they are on our side] – but that’s not the point, is it? It’s the government you’re dealing with’. Overall, these findings show that on a deliberative level, CSOs not only need to strategically frame their demands so as to align with the discourse of the dominant party – they also need to frame them in ways consonant with opposition parties’ discourse in order to facilitate broad-based, cross party engagement and lessen dominant party institutionalisation, as well as overcome cognitive locks.

### CSO networking and alliance building

This section of the findings applies theory on policy networks, CSO alliance building and neo-corporatism to reveal how, under OPD, the disproportionate power of the dominant party distorts collective action and leads to ‘defensive’ networking amongst CSOs in order to counter the hegemony of the governing party. The theoretical backdrop to this is that networks and mobilising structures sustain collective action in civil society (McAdam et al., 1996: 13). Taken together, they are the means for interorganisational coordination and collaboration between CSOs. Interviewees’ comments reveal how participatory mainstreaming is affected by single-party dominance. Specifically, the fact that the dominant party is unlikely to be voted out of office diminishes its propensity to compromise its policy agenda in the face of CSOs’ mainstreaming claims. In turn, this may further weaken and disadvantage ‘standalone’ organisations who, even in ‘regular’ liberal democracies, are often easier for political elites to sideline or defeat compared to coordinated action from multiple networked CSOs (cf. Banazak, 2010). The disproportionate power of the dominant party under OPD strengthens the incentives for CSOs to effectively seek ‘strength in numbers’ and coordinate when engaging with government (Sabatier and Jenkins-Smith, 1993). This is evident in the present study. For example, an interviewee alluded to how CSOs representing individual equalities ‘strands’ (e.g. gender, disability, ethnicity and so on) had decided to unite to produce a manifesto of equalities demands spanning protected characteristics. The underlying rationale was that it would afford them greater influence over the dominant party’s programme in the run-up to national elections:

We’d seen what had happened on [the previous election commitment by the dominant party to] ‘free’ social care [the party did a U-turn, widely seen as reneging] … this was a terrible betrayal of disabled people … We thought right! So, yes, we did sign-up to the [Equalities] Manifesto … our Exec[utive] Committee said we must make sure that it [government ‘betrayal’] doesn’t happen again, so having everyone on-board [i.e. coordinated claims-making by CSOs] ticks that box … shifts the balance … [of power with the governing party].

The current study also reveals the impact of single-party dominance and neo-corporatism. As Mansbridge (1999: 495) explains, the latter as an approach that ‘values interest groups as ongoing institutional mechanisms for representing interests not easily represented in the territorial representative process. [And] it attempts to bring the *laissez faire* system of interest representation partly under public control’. In the current research context, neo-corporatism takes the form of state-sponsored policy networks. Whilst undoubtedly facilitating engagement, its use can also be emblematic of the coercive aspect of mainstreaming (cf. Payne, 2011: 528).

Accordingly, CSOs’ proximity to power elites under neo-corporatism may undermine their autonomy and criticality, thereby compromising their independence (Korolczuk, 2014; Mansbridge, 1999). Thus, as De Jager (2005: 56) explains:

civil society derives its very legitimacy from its ability to act and then to act independently … The development of more formal and regulated civil society–state relations may subvert the character of civil society and compromise its role in enhancing democracy.

Whilst this concern is germane to liberal democracies in general, interviewees’ comments attest to its heightened significance under OPD because of the increased power of political patronage stemming from power centred on a single party.^11^ Thus, the director of a CSO reflected: ‘we don’t want to alienate [ministers] but also we don’t want to be in the pocket of the minister and it’s that balance which is difficult’. Another offered candidly: ‘we are afraid to say “the emperor has no clothes”^12^ … we are afraid to show when failure is happening, because you know you have several people that have to wear several hats [i.e. perform multiple roles as CSO representatives and members of government policy task and finish groups, and recipients of state funding], there *are* conflicts of interest … ’Another manager alluded to how OPD even limited CSOs’ willingness to provide information to assist opposition backbenchers in criticising government policy. This is because its specialist nature meant its provenance would be clear (and in turn likely to be viewed as an overt attack on government, their chief sponsor). One manager said, ‘it can misfire … having the right points advanced by the “wrong party”’ – er, [irony] it doesn’t always advance your cause!’. Another reflected on the overall impact of OPD and neo-corporatist practices:

In effect you now have state sponsored civil society which compounds the problem that it’s hard to criticise government. So they’ve created a civil society but it’s an anaemic one because they control the flow of blood and they don’t want it [civil society] to get too strong.

Whilst extant work often points to political parties and civil society as largely discrete entities (cf. Chatterjee, 2004: 136; Kopecky and Mudde, 2003), single-party dominance highlights blurred boundaries (cf. Mair, 2000) and how this may impact on participative mainstreaming. Under OPD holding power over successive election cycles effectively institutionalises the dominant party. It regularises and sustains contacts between government and civil society over extended periods of time (‘boundary blurring’) – whereas such relationships and interaction would be swept away by a new party being elected to power in regular turnover democracies. As Bénit-Gbaffou (2012: 186) explains, the resultant overlapping of government and civil society spheres affects participative mainstreaming by limiting CSOs’ criticality towards the governing party. This can be prompted by a range of reasons – including citizens’ loyalty to the party as well as a shared sense of identity and/or ideology. It may also be due to the dominant party ‘exert[ing], through its members, a subtle but efficient social control on what is said … it may [also] be shaped by everyone’s perception that their discourse and position might be reported to [party] structure’ (Bénit-Gbaffou, 2012: 191).

The latter point is supported by the present findings for interviewees spoke of ‘toning down’ or avoiding criticism of government for fear that some CSO managers – who were also members of the dominant party, convey their comments to ministers; which in turn could jeopardise their government funding. According one, “you do have to stop yourself. … I thought hold-on [uses own name] watch yourself! ‘cos I know a couple of policy people there are strong Party [people]”. A further, related issue is that mainstreaming may be undermined by the negative aspects (opaqueness, lack of accountability and exclusive nature) of informal networks that crosscut formal political channels and processes (Masket, 2009). This corresponds with existing work by McClurg and Lazer (2014: 4) who explicate how

party organizations, interest groups and others in the policy process … often form connections that are at times fluid and at other times enduring … suggest[ing] a host of new substantive questions about organized influence in politics that centred on explaining relationship formation and impact in political organizations.

Thus, interviewees described how critical engagement with government routinely took place outside legislative channels. In other words, OPD was “short circuiting” the formal practices of parliamentary representation and instead operating as an informal, intraparty process. One interviewee said: ‘we are not going in from a zero baseline. Our existing contacts and connections are very important. There are ‘soft targets’ in the [National] Assembly’. Another echoed this by making the point that, ‘you have to be in the right groups or else you will get marginalised. You have to network’. According to another CSO manager, “some of the civil service they are very much steeped in the old ways, the Whitehall approach … and that’s where we’ve had to make representations, you know, through political channels … ”Another added:

I found where we have had some successes is in raising concerns with Labour backbenchers who have gone on to raise the concerns themselves with ministers. So we tend to shape the policy practices indirectly through the Labour Party machine itself, shaping policy internally. I think there’s more chance of a Labour backbencher getting concessions out of a [Labour] minister than engaging opposition parties “from the outside.”

A further issue is the way that OPD may increase division and mistrust between equalities CSOs. This is because the greater powers of government patronage in OPD systems may create two classes of CSO – ‘insiders’ and ‘outsiders’ (Taylor, 2001), thereby accentuating conflict and tensions between equalities strands (e.g. Siim, 2007). Thus, for example, one CSO chief executive said

we have this situation where – when it comes to disabled people [named organisation] are widely seen by the minister as *the* representative body … Something, obviously, they have done nothing to disabuse … We may be [a] small [CSO] but … I mean, effectively, y’ know we are crowded out … 

In a broadly similar vein, another interviewee said

it all really comes down to *who* speaks for [ethnic minority] communities … now [named umbrella body] are funded by the government, right? They know how to play the game … they’re not going to press the minister hard; truth told, they are not going to press the minister! [laughs] … [named equality body] said ‘why aren’t you members’? [of the umbrella body]. Look, there’s no way our people would have it. We’re *not* becoming part of that charade.

In summary, the practical significance of these findings for CSOs is that in placing their equalities claims on government they need to utilise mobilizing structures to develop advocacy coalitions that act to counter the disproportionate power and influence of dominant parties. Such alliances offer a way to address the compromised independence that can result from dominant party patronage and neo-corporatist structures. In other words, by creating alliances that are broader than state-sponsored CSOs, full criticality may be restored.

## Conclusion

The foregoing empirical analysis reveals that single-party dominance influences CSOs’ engagement strategies by shaping the prevailing political opportunity structures and attendant CSO strategies for participation in the work of government. Instead of adapting their *Modus operandi* to take account of governing party turnover as happens in ‘regular’ liberal democracies, the current data show how single-party dominance distorts civil society policy engagement. The analysis identifies four areas affected by the absence of turnover: strategic bridging, action repertoires, framing and cognitive locks, and CSO networking and alliance building. Thus, interviewees’ comments attest to how OPD skews the ‘political dynamic’ for participation by affecting group incentives. Moreover, attention to strategic bridging reveals how single-party dominance influences whom to engage amongst the political elite. Whilst reference to CSOs’ action repertoires also reveals how some view the absence of turnover as a pathology that limits policy input. In addition, the application of social theory on framing and ‘cognitive locks’ illustrates how, under OPD, CSOs are more constrained in their use of language. Rather than a free exchange of ideas, interviewees speak of deliberately advancing claims in ways that align with dominant party thinking. Furthermore, conceptual work on policy networks and neo-corporatism underlines how the disproportionate power of the dominant party distorts collective action and leads to ‘defensive’ networking amongst CSOs in order to counter the hegemony of the governing party.

Thus, far from being an apolitical, bureaucratic, instrumental undertaking, the operationalisation of mainstreaming principles around participation and democracy is shown to be shaped by electoral politics. As the principal policy actor bound by international and domestic mainstreaming obligations, government is not a constant in the mainstreaming equation. Rather, its political complexion changes each time a governing party is voted out and replaced by a rival. Accordingly, the type of party holding office shapes a broad range of factors including: the power of the incumbent, the degree of openness or resistance to exogenous criticality and claims making, the extent of political patronage and the degree of CSO resource dependency and autonomy.

A key point emerging from the study is that the nondiscrete issues identified are self-sustaining. Over time, diminished civil society engagement with opposition parties will further strengthen the dominant party and thus perpetuate a downward spiral such that a change of governing party is even less likely. This provides a salutary lesson for the 180 + liberal democracies subscribing to mainstreaming. To counter such problems, CSOs’ need to employ adaptive action repertoires for participative mainstreaming. These should be broad based and cross-party in nature. For example, they may include the modification of strategic bridging to compensate for OPD through deliberate engagement with weaker opposition parties. Moreover, they may use their mobilising structures to develop advocacy coalitions to counter the compromised independence that can result from dominant party patronage and neo-corporatist structures. Through such measures, full criticality in state–civil society relations may be restored. Overall, the wider contribution of this study to understanding civil society engagement is threefold: (1) It reveals the types of problems that may emerge in relation to PDM when a governing party retains power over several electoral cycles, (2) It underlines the need for contemporary academic analysis to be cognizant of how Participatory Democratic Mainstreaming is contingent on electoral competition and governing party turnover, and (3) As noted, it emphasises that CSOs’ need to employ an adaptive model of policy engagement suited to the prevailing electoral politics and governing party turnover in liberal democracies.

## Notes

1 Council Directive 2004/113/EC of 13 December 2004 implementing the principle of equal treatment between men and women in the access to and supply of goods and services http://eur-lex.europa.eu/legal-content/en/ALL/?uri=CELEX:32004L0113 and http://eur-lex.europa.eu/legal-content/EN/TXT/?uri=CELEX:32006L0054 (accessed 2 January 2014).

2 See section 4, http://www.legislation.gov.uk/sdsi/2011/9780111012215/contents (accessed 20 January 2015).

3 2001/41 of 26 July 2001, 2002/23 of 24 July 2002, 2003/49 of 24 July 2003, 2004/4 of 7 July 2004, 2005/31 of 26 July 2005, 2006/36 of 27 July 2006, 2007/33 of 27 July 2007, 2008/34 of 25 July 2008 and 2009/12 of 28 July 2009.

4 E.g. Section 3, Equality Act 2010 (Specific Duties) (Scotland) Regulations 2012. http://www.legislation.gov.uk/sdsi/2012/9780111016718/contents (accessed 12 October 2014).

5 In coalition 2000–2003 and 2007–2011, with the Liberal Democrats and Plaid Cymru, respectively.

6 Under Economic and Social Research Council Awards No: R000239410 and L219252016.

7 Thus, engagement and governing party turnover are the dependent and independent variables, respectively.

8 i.e. Systems with free and fair elections.

9 ‘Groundhog Day’ – a 1993 film directed by Harold Ramis concerning a time loop – cited here as a simile for repetition.

10 In this sense, the present findings echo the experience elsewhere. For example, in Bavaria where, even with direct democracy mechanisms such as local referendums, limited turnover of the governing party is amongst factors that limit the agenda setting of CSOs (see Premat, 2012: 169).

11 In this respect, the findings resonate with potential issues in Bavaria, another ‘regional’ European polity characterised by limited governing party turnover where government has invested significant political capital in developing state–civil society engagement (cf. Raffl, 2014).

12 Reference to Hans Christian Andersen story – here meaning CSOs were afraid to be critical of government.
